# Enrichment of rare *CFTR* variants in Finnish patients with congenital chloride diarrhea

**DOI:** 10.1371/journal.pone.0318249

**Published:** 2025-02-24

**Authors:** Satu Wedenoja, Jarmo Ritari, Jukka Partanen, Juha Kere, Kaija-Leena Kolho

**Affiliations:** 1 Information Services Department, Finnish Institute for Health and Welfare, Helsinki, Finland; 2 Obstetrics and Gynecology, University of Helsinki and Helsinki University Hospital, Helsinki, Finland; 3 Stem Cells and Metabolism Research Program, University of Helsinki, Helsinki, Finland; 4 Folkhälsan Research Center, Helsinki, Finland; 5 Finnish Red Cross, Blood Service, Helsinki, Finland; 6 Department of Biosciences and Nutrition, Karolinska Institutet, Huddinge, Sweden; 7 Children’s Hospital, Pediatric Research Center, University of Helsinki and Helsinki University Hospital, Helsinki, Finland; Shaheed Rajaei Cardiovascular Medical and Research Center: Rajaie Cardiovascular Medical and Research Center, IRAN, ISLAMIC REPUBLIC OF

## Abstract

**Objective:**

The autosomal recessive disease congenital chloride diarrhea (CLD), caused by loss-of-function mutations in the *solute carrier family 26 member 3* (*SLC26A3*) gene, shows association with inflammatory bowel disease (IBD). However, it is unclear whether IBD risk is associated with genetic or immune signatures. SLC26A3 interacts with several ion transporters linked to intestinal inflammation, such as cystic fibrosis transmembrane conductance regulator (CFTR) and solute carrier family 9 member 3 (SLC9A3) causing congenital sodium diarrhea. We hypothesized that other epithelial channels affecting intestinal salt balance might modulate CLD phenotype or IBD risk.

**Materials and methods:**

We analyzed 495 gene variants within 33 ion transporters among 28 patients with CLD and 44,443 population controls.

**Results:**

We found three intronic variants at or near the *CFTR* locus (rs17132543, rs2283054 and rs76622533) showing statistically significant (*P* < 1.42x10^-5^) associations with CLD.

**Conclusions:**

These data demonstrate enrichment of rare variants at the *CFTR* locus in chromosomes harboring the Finnish founder mutation for CLD.

## Introduction

Congenital chloride diarrhea (CLD; OMIM #214700) is an autosomal recessive disease caused by mutations in the *solute carrier family 26 member 3 (SLC26A3* alias *DRA*) gene [1]. Impaired SLC26A3-mediated chloride-bicarbonate transport in CLD, and the coupled failure of sodium-hydrogen exchanger 3 (NHE3 alias SLC9A3) function, disrupt sodium chloride and fluid reabsorption in the terminal ileum and colon [2]. While diarrhea is chronic, early diagnosis and sufficient salt substitution therapy provide a favorable long-term outcome in CLD [3].

Both patients with CLD and *slc26a3*-deficient mice have an elevated risk of inflammatory bowel disease (IBD) [4,5]. To support the primary role of SLC26A3 in the pathogenesis, *SLC26A3* polymorphisms and downregulation have been linked to intestinal inflammation in non-CLD subjects [6,7] and no changes in fecal microbiota characterize CLD-associated IBD [8]. As IBD is found in 17% of adolescent and adult patients with CLD [4], however, factors other than the *SLC26A3* genotype only are likely to promote intestinal inflammation.

Remarkably, other ion transporters interacting with SLC26A3 show associations with IBD. In the ileum and proximal colon, absorption of sodium chloride occurs predominantly via the coupled action of SLC26A3 and SLC9A3 [2], which is disrupted in congenital sodium diarrhea (CSD) and linked to IBD risk [9]. As for intestinal secretion, chloride channel cystic fibrosis transmembrane conductance regulator (CFTR), associated with intestinal inflammation [10], specifically up-regulates anion exchange activity of the SLC26 transporters including SLC26A3 [11].

When combined with the crucial role of several ion transporters in intestinal inflammation [12], these data prompted us to study whether other epithelial transporters affecting intestinal salt balance show association with CLD. To address this question, we utilized the unique series of Finnish patients with CLD and large number of population controls, and analysed 495 gene variants of 33 ion transporters for possible association with CLD.

## Methods

We utilized DNA samples extracted from buccal swabs from 28 subjects of our clinical series of CLD, originally collected to study the association between CLD and IBD [8]. Characteristics of the study series have been described previously [8]. All of them were homozygous for the Finnish founder mutation V318del of the *SLC26A3* gene [1]. They had regular salt substitution therapy with a median dose of chloride 3 mmol/kg/day, being compatible with the recommendation of 3-4 mmol/kg/day [3,8]. Two of the subjects included in this study had IBD. The series was collected between 16 March and 19 December 2018, and supervised by the Children´s Hospital. Study protocol was approved by the ethics committee of the Hospital District of Helsinki and Uusimaa (HUS/895/2017). All subjects, or their legal guardians, gave their signed informed consent as waived by the ethics committee. Research was performed in accordance with the Declaration of Helsinki and in accordance with the relevant guidelines and regulations.

The CLD cases (n = 28) were genotyped on the Thermo Fisher Axiom FinnGen2.r4 genotyping array at Eurofins Genomics Europe Genotyping A/S (www.eurofinsgenomics.eu) (Aarhus, Denmark). The genotypes of 44,443 Finnish blood donors were obtained from the Blood Service Biobank of the Finnish Red Cross Blood Service, Vantaa, Finland. The Biobank blood donors have given a biobank consent according to the Finnish Biobank Act. The present study has biobank permit 002-2020-5. The blood donors were genotyped as part of the FinnGen project [13] on the FinnGen array (https://www.finngen.fi/en/researchers/genotyping) and returned to the Blood Service Biobank. All study subjects provided a written informed consent. The genotype data were managed and analyzed with PLINK v1.90b6.24 64-bit (6 Jun 2021) (www.cog-genomics.org/plink/1.9/) [14], PLINK v2.00a5.7LM AVX2 Intel (30 Oct 2023) (www.cog-genomics.org/plink/2.0/) [14], and R software v4.3.3 [15].

Gene coordinates of 40 target genes in GRCh38 genome build, selected based on their roles in intestinal ion transport or SLC26A3 interaction, were extracted from the ENSEMBL database release 110 [16]. To analyze the genetic variants in the 40 target genes, the SNPs located in these genes were identified based on the coordinates and extracted from the genotype data using the plink2 command *--extract*. In total, 704 SNPs were found within 33 of 40 target genes.

As both cases and controls were genotyped on the same array platform, the data could be merged variant-by-variant without allele orientation or strand discrepancies using the plink1.9 command *--bmerge*. Genetic principal components (PC) were computed on the merged genotype data with the plink2 command *--pca approx* using 22,774 autosomal biallelic variants selected randomly from all over the genome and having minor allele frequency of at least 12% to exclude too rare genotypes from PC analysis in the CLD cases. As the number of cases was relatively low in our study, we used a minor allele frequency (MAF) threshold of 12% to achieve a sufficient statistical power.

CLD cases vs. controls association analysis was performed using the plink2 command *--glm* which fits a logistic regression model with Firth’s correction [17] on the genotypes and binary phenotype data. The first ten genetic principal components were included as model covariates with the command *--covar*. Owing to the significant case/control imbalance (~1/1600), a minimum minor allele count of 800 was imposed on the analysis using the plink2 command *--mac 800* to ensure that the firth’s correction remains calibrated (https://www.cog-genomics.org/plink/2.0/assoc#glm). Total of 495 variants fulfilled this condition. Follow-up analysis with adjusting for the *CFTR* lead SNP rs76622533 genotype was performed as described above except for adding the rs76622533 genotype as an additional covariate in the model using the plink2 command *--condition*. The association results data were plotted with the R library ggplot2 v3.4.2 [18]. The P value threshold was set as Bonferroni correction at the level of 0.01, separately for both association runs. In the first run, CLD vs. controls, the P value threshold was 2.02x10^-5^ (0.01/495). In the second run, CLD vs. controls adjusted for rs41280236, the p-value threshold was 2.32 x10^-5^ (0.01/448). There were fewer tests in the second run due to some SNPs failing in the Plink association analysis after the adjustment. Considering the number of analysed variants and low number of cases, P values < 1.42x10^-5^ were considered statistically significant.

## Results

We compared the genotypes of 28 CLD subjects with those of 44,443 blood donors. We found three SNPs for *SLC26A3* and three SNPs for the expanded *CFTR* locus, including *Wnt Family Member 2* (*WNT2*) gene, with significantly different patterns of frequencies among CLD cases vs. controls (Table 1; S1 Table).

**Table 1:** Gene variants showing association with congenital chloride diarrhea.

Chromosome	Position	rsID	Gene	Location	Reference allele	Alternative allele	Alternative allele frequency, CLD cases	Alternative allele frequency, controls	P value
7	107795787	rs10953548	*SLC26A3*	Intron 1	G	A	0.04	0.45	1.26x10^-6^
7	107800728	rs1347294065	*SLC26A3*	Intron 1	GT	G	0.04	0.42	3.44x10^-6^
7	107783462	rs41280236	*SLC26A3*	Intron 8	T	C	0.93	0.15	3.38x10^-21^
7	117292480	rs17132543	*WNT2*	Intron 4	A	G	0.32	0.09	2.23x10^-9^
7	117486347	rs2283054	*CFTR*	Intron 1	G	A	0.32	0.09	1.31x10^-9^
7	117543084	rs76622533	*CFTR*	Intron 9	G	T	0.30	0.05	2.88ex10^-16^

For *SLC26A3*, we found alternative allele enrichment for the leading SNP rs41280236 (intron 8) of 0.93 in CLD vs. 0.15 in controls (P value 3.38x10^-21^). For the other two *SLC26A3* SNPs, located in intron 1, both the rs10953548 alternative allele (P value 1.26x10^-6^) and the rs1347294065 alternative allele (P value 3.44x10^-6^) were rare with the frequency of 0.04 in CLD compared with the frequencies of 0.45 and 0.42 in controls, respectively.

As for *CFTR* locus, alternative alleles showed increased frequency of 0.32 in CLD vs. 0.09 in controls for rs2283054 (intron 1; P value 1.31x10^-9^), and 0.30 in CLD vs. 0.05 in controls for rs76622533 (intron 8; P value 2.88x10^-16^). Moreover, alternative allele for rs17132543, located in the intron 4 of the *WNT2* gene upstream of *CFTR*, showed increased frequency of 0.32 in CLD vs. 0.09 in controls (P value 2.23x10^-9^).

**Fig 1:**
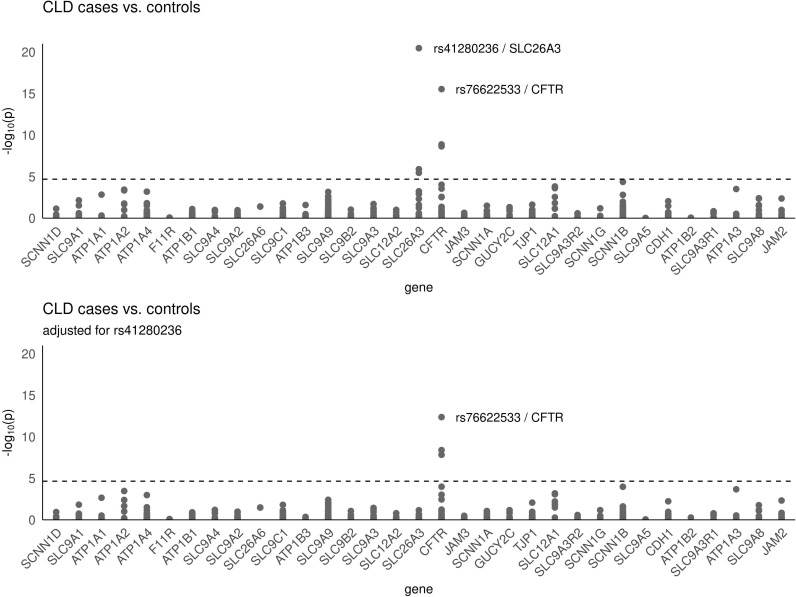
Manhattan plots of study results. The results of SNP association analysis for 495 variants within 33 genes are shown for all subjects. The horizontal dotted line indicates the significance threshold (*P* < 1.42e-05). CLD indicates congenital chloride diarrhea.

We further adjusted the data for the leading SNP of *SLC26A3* (rs41280236). We found that the two other *SLC26A3* were in linkage disequilibrium to the leading SNP, because adjusting for rs41280236 removed their signals (Fig 1). In contrast, adjusting for rs41280236 of *SLC26A3* did not remove the signals for significant SNPs at the *CFTR* locus, indicating that *CFTR* association is independent of *SLC26A3* (Fig 1)*.*

## Discussion

This study demonstrates an *SLC26A3-*independent association between CLD alleles and three variants of the *CFTR* locus. We found no similar associations for the other ion transporters except for the disease gene *SLC26A3,* for which a strong intronic signal emerged in intron 8, close to the Finnish founder mutation for CLD, and weaker signals arose in intron 1. These data indicate that despite the homozygous founder mutation responsible for almost all cases of CLD in Finland [1], some recombination events have occurred for the ancestral V318del allele at the *CFTR* locus.

A main strength of our study is the CLD series that is one of the largest globally [3]. An additional strength is the large population control data of blood donors, through which the *CFTR* locus association was found. The main limitation of our study is, however, that the SNPs we found are non-coding and their role for CFTR function remains obscure. Moreover, we had only two cases with CLD-associated IBD here and it was not possible to study whether this phenotype shows association with *CFTR*-related variants. Due to the overall low number of study cases (n = 28) in our data, we saw it necessary to use a MAF threshold of 12% to reach a sufficient statistical power. At MAF 5%, there would not have been hardly any heterozygotes among the cases.

Although no clear phenotypic differences exist for the gut features in the V318del homozygous CLD patient population [3], only 17% of adult and adoslescent patients have developed IBD [4]. Our study shows that the majority of V318del chromosomes are highly similar, consistent with the founder effect and a single ancestral origin of this allele in the population. The rs41280236*C variant in intron 8, showing the allele frequency of 0.93 in CLD, seems to be in a tight linkage disequilibrium to the founder mutation located in the neighboring exon 8 of the *SLC26A3* gene. Otherwise, these results indicate that the V318del homozygous population is highly homogenous for *SLC26A3* structure, with the majority of variation being individual or due to low-frequency variants within intron 1 (rs10953548 and rs1347294065), showing enrichment of reference alleles in CLD but not overlapping with known regulatory elements.

As for CLD subjects, two variants showed enrichment within *CFTR* introns and one upstream of the expanded *CFTR* locus at the *WNT2* gene. The leading SNP rs76622533 in intron 9 of *CFTR*, and rs2283054 in intron 1, are not located close to splicing sites, and database searches provide no support for clinical association. The third SNP rs17132543, located in the intron 4 of the neighbouring gene *WNT2,* lies outside but close to CFTR’s known regulatory elements [19].

Originally, the positioning of CLD gene in chromosome 7 was based on a candidate gene idea: could *CFTR* also be a gene for CLD? That the CLD patients were not homozygous for *CFTR* markers, as expected in a recessive disease, ruled out the role of *CFTR* in the pathogenesis. However, the observed strong linkage disequilibrium between *CFTR* markers and CLD revealed that the disease gene must be very close. Fine mapping with microsatellites showed that 45% of the Finnish CLD chromosomes have recombined, with around 11 historical recombinations, between *SLC26A3* and *CFTR* [20]. Thus, our results are in agreement with the earlier findings of recombination and linkage disequilibrium between *SCL26A3* and *CFTR* in CLD.

Because *CFTR* and *SLC26A3* are physically close to each other on chromosome 7, their causal and non-causal variants may correlate as a result of linkage disequilibrium, making direct biological inferences complicated. However, the association between the *CFTR* locus and CLD in this study was *SLC26A3*-independent. Due to the reciprocal activation of SLC26 transporters and CFTR (11), their connection in a genomic context is interesting, even if we have no functional data linking the intronic *CFTR* SNPs to CLD or genotype-phenotype relationship.

Collectively, these data suggest that the Finnish CLD chromosomes harboring the founder mutation V318del are not completely identical. While overall variation is rare among ion transporters, rare recombination events have occurred at the *CFTR* locus in CLD-related chromosomes.

## Supporting information

S1 Table Association of ion transporters with CLD.
(XLSX)
